# Direct association between rainfall and non-typhoidal *Salmonella* bloodstream infections in hospital-admitted children in the Democratic Republic of Congo

**DOI:** 10.1038/s41598-021-01030-x

**Published:** 2021-11-03

**Authors:** Bieke Tack, Daniel Vita, Marie-France Phoba, Lisette Mbuyi-Kalonji, Liselotte Hardy, Barbara Barbé, Jan Jacobs, Octavie Lunguya, Liesbet Jacobs

**Affiliations:** 1 Department of Clinical Sciences, Institute of Tropical Medicine, Antwerp, Belgium; 2 Department of Microbiology, Immunology and Transplantation, KU Leuven, Leuven, Belgium; 3 Saint Luc Hôpital Général de Référence Kisantu, Kisantu, Democratic Republic of Congo; 4 Department of Microbiology, Institut National de Recherche Biomédicale, Kinshasa, Democratic Republic of Congo; 5 Department of Medical Biology, University Teaching Hospital of Kinshasa, Kinshasa, Democratic Republic of Congo; 6 Department of Earth and Environmental Sciences, KU Leuven, Heverlee, Belgium; 7 Ecosystem & Landscape Dynamics, Institute for Biodiversity and Ecosystem Dynamics, University of Amsterdam, Amsterdam, The Netherlands

**Keywords:** Bacteriology, Clinical microbiology, Environmental impact, Infectious diseases, Epidemiology, Paediatric research

## Abstract

Non-typhoidal *Salmonella* (NTS) ranks first among causes of bloodstream infection in children under five years old in the Democratic Republic of Congo and has a case fatality rate of 15%. Main host-associated risk factors are *Plasmodium falciparum* malaria, anemia and malnutrition. NTS transmission in sub-Saharan Africa is poorly understood. NTS bloodstream infections mostly occur during the rainy season, which may reflect seasonal variation in either environmental transmission or host susceptibility. We hypothesized that environment- and host-associated factors contribute independently to the seasonal variation in NTS bloodstream infections in children under five years old admitted to Kisantu referral hospital in 2013–2019. We used remotely sensed rainfall and temperature data as proxies for environmental factors and hospital data for host-associated factors. We used principal component analysis to disentangle the interrelated environment- and host-associated factors. With timeseries regression, we demonstrated a direct association between rainfall and NTS variation, independent of host-associated factors. While the latter explained 17.5% of NTS variation, rainfall explained an additional 9%. The direct association with rainfall points to environmental NTS transmission, which should be explored by environmental sampling studies. Environmental and climate change may increase NTS transmission directly or via host susceptibility, which highlights the importance of preventive public health interventions.

## Introduction

**Figure 1:**
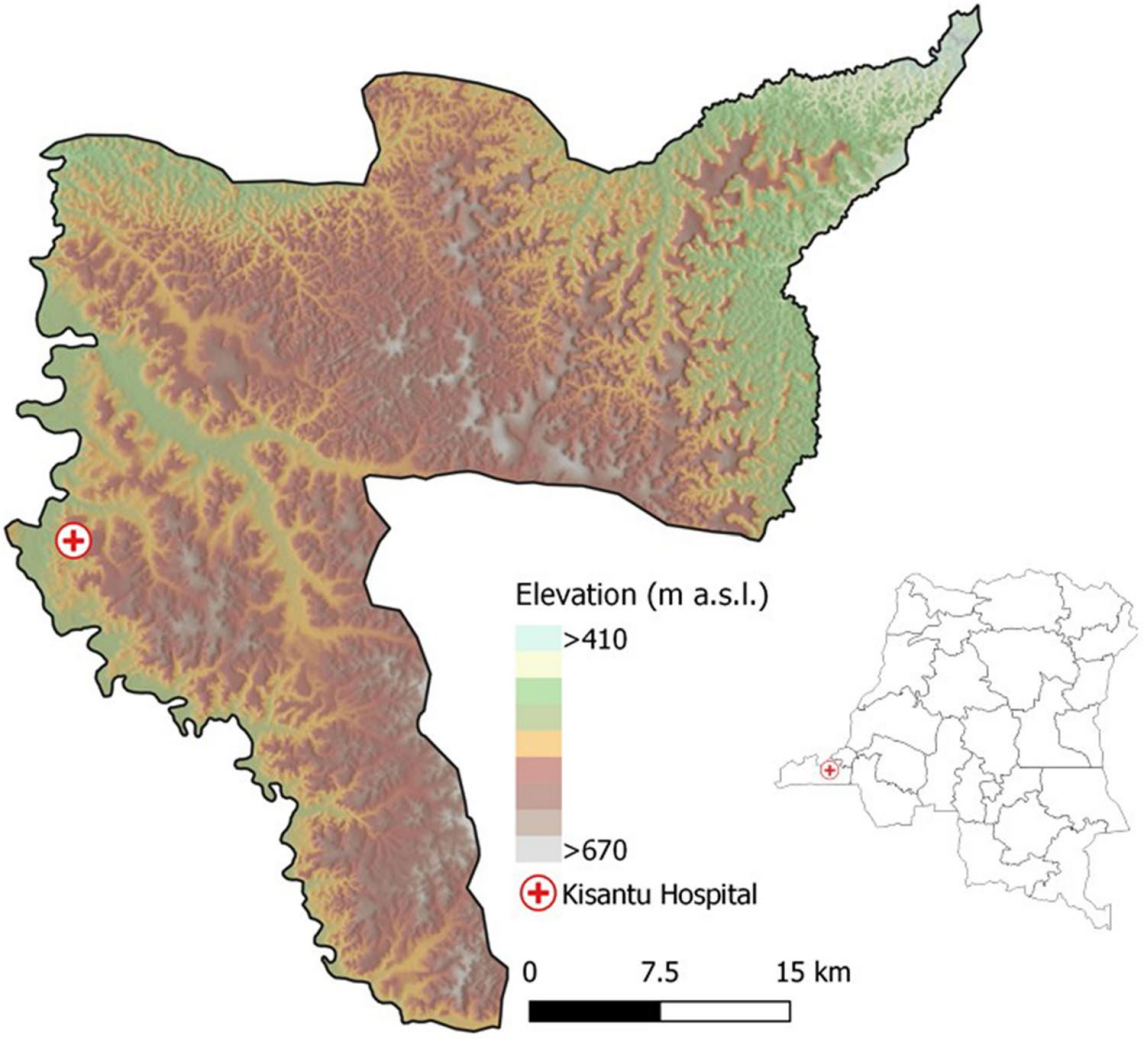
Geographical location of Kisantu Hospital in Kisantu health district in the Democratic Republic of Congo. Maps were made in QGIS 3.4 and geographical data on the administrative boundaries of Kisantu health zone and location of Kisantu hospital were shared by the Belgian development aid agency (Enabel)^12^, provinces were based on data shared by the Royal Museum of Central Africa (Tervuren, Belgium), elevation and hill shade are based on the SRTM V4, 3 arcsec^13^.

Non-typhoidal *Salmonella* (NTS) disease is a leading cause of bloodstream infection in sub-Saharan Africa. It was estimated that 421,600 cases of NTS disease occurred in sub-Saharan Africa in 2017^1^, accounting for 49,440 deaths^1^. Due to its high case fatality rate (15.8%)^1^, its non-specific clinical presentation^2,3^ and its frequent antimicrobial resistance^2–4^, NTS disease is a major public health problem. The burden of NTS is concentrated in children under five years old with risk factors being *Plasmodium falciparum* (*Pf*) malaria, anemia and malnutrition, and in HIV infected individuals^1–3^. In the Democratic Republic of Congo (DR Congo), the NTS burden is immense and remains high^5^. In children under five years old admitted to Kisantu general referral hospital (Kisantu hospital; Kongo Central Province, Fig. 1), NTS were isolated in 3 out of 4 culture confirmed bloodstream infections^5^.

The reservoir and transmission of NTS in sub-Saharan Africa remains unclear^2,3^. In high income countries, the NTS reservoir and transmission are zoonotic and foodborne^3^. However, in sub-Saharan Africa, NTS may have adapted to the human host and have a human reservoir^2,3,6,7^. In analogy to *Salmonella* Typhi, the human-restricted cause of typhoid fever, transmission of NTS in sub-Saharan Africa may occur through food and water contaminated with human feces^2,3^. Such waterborne environmental transmission could be further aggravated by poor sanitation, poor access to clean water and poor hygiene^3^. Intense rainfall causing flooding and water run-off can cause contamination of surface water and increase dispersal of *Salmonella* species in the environment^8–10^. Moreover, temperature may influence environmental multiplication and survival of *Salmonella* species^8–10^.

In sub-Saharan Africa, NTS bloodstream infections are more prevalent during the rainy season^3,11^. Similarly, the host-associated factors *Pf* malaria, anemia and malnutrition are also more frequent during the rainy season^3,11^. We therefore hypothesize that NTS seasonal dynamics are driven by a complex interaction of environment- and host-associated factors, which nevertheless contribute independently to NTS seasonal dynamics. We hypothesize that environment-associated factors not only increase host susceptibility, but may also increase environmental NTS contamination during the rainy season. A better understanding of environmental drivers will provide insights into the potential environmental NTS transmission pathways and the potential impact of environmental change, and will orient control measures.

In this retrospective study, we aimed to explain the seasonal dynamics of NTS bloodstream infections in children under five years old admitted to Kisantu hospital. We used open-access remotely sensed rainfall and temperature data as proxies for environment-associated factors and principal component analysis to disentangle them from host-associated factors. We used timeseries regression to assess their independent association with NTS seasonal dynamics.

## Results

### Description of the seasonal dynamics of NTS and its environment- and host-associated factors

**Figure 2:**
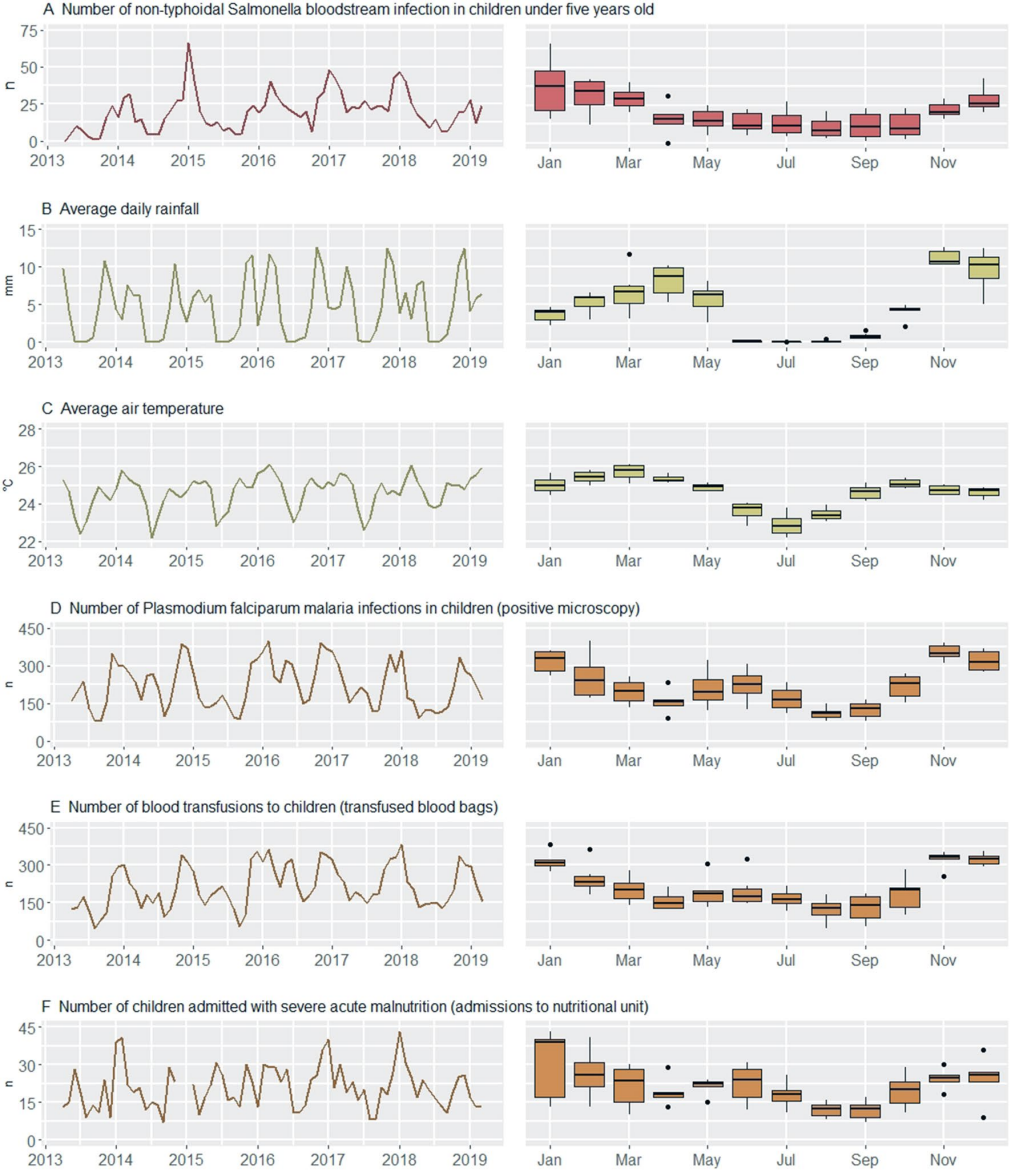
Timeseries (left) and seasonality (right) plots of non-typhoidal *Salmonella* (NTS) bloodstream infections in young children (red), of environment- (green) and host-associated (brown) factors for NTS bloodstream infection. In both plots, the NTS infections and host-associated factors are expressed as number of cases per month, and the environment-associated factors as the average of the daily rainfall and average of the air temperature per month. The seasonality plot is based on the full data period.

During the six-year study period (2013–2019), 1434 (9.8%) NTS were isolated out of 14,598 blood cultures from children between 28 days and five years old. There was a gradual increase in the number of isolated NTS (factor 2.9 increase) and sampled blood cultures (factor 1.9 increase)per year between 2013 and 2018 (Supplementary Figs. S1 and S2E). *Pf* malaria infection was confirmed in 15,917 (49.1%) out of 32,422 microscopy examinations in children. Between 2013 and 2018, a 1.2 factor increase in malaria microscopy examinations per year was observed with a 1.1 factor increase in confirmed *Pf* malaria infections per year (Supplementary Figs. S1 and S2E). Over the six years, 15,374 blood bags were transfused to children and 1451 children with severe acute malnutrition were admitted to the nutritional unit. Data on the number of children admitted with severe acute malnutrition were missing between December 2014 and January 2015. Most NTS (79.8%) were isolated during the wet months October–May with highest numbers of NTS bloodstream infections (median = 20–38 NTS infections per month) observed from November till March. Similarly, there were more *Pf* malaria infections (median = 199–348 *Pf* malaria infections per month) and blood transfusions during these months. Although the seasonal dynamics of children admitted with severe acute malnutrition were more variable, most (73.4%, underestimation due to missing data December 2014–January 2015) of these admissions occurred during wet months. (Fig. 2).

Climate Hazards Group InfraRed Precipitation with Station Data (CHIRPS) rainfall data showed a clear seasonal pattern with the most intense rainfall in November and December (average daily rainfall 10–13 mm/day) and a dry period from June to August. The average total rainfall per year was 1688 mm. The monthly average air temperature (ERA5) fluctuated around 25 °C during wet months and around 23 °C during dry months. Coldest days occurred in the dry months June–August with monthly minimum daily average temperatures between 20 and 22 °C. The hottest days were observed between February and April with monthly maximum daily average air temperature at 26–27 °C.

### Correlation between NTS and its environment- and host-associated factors

**Figure 3:**
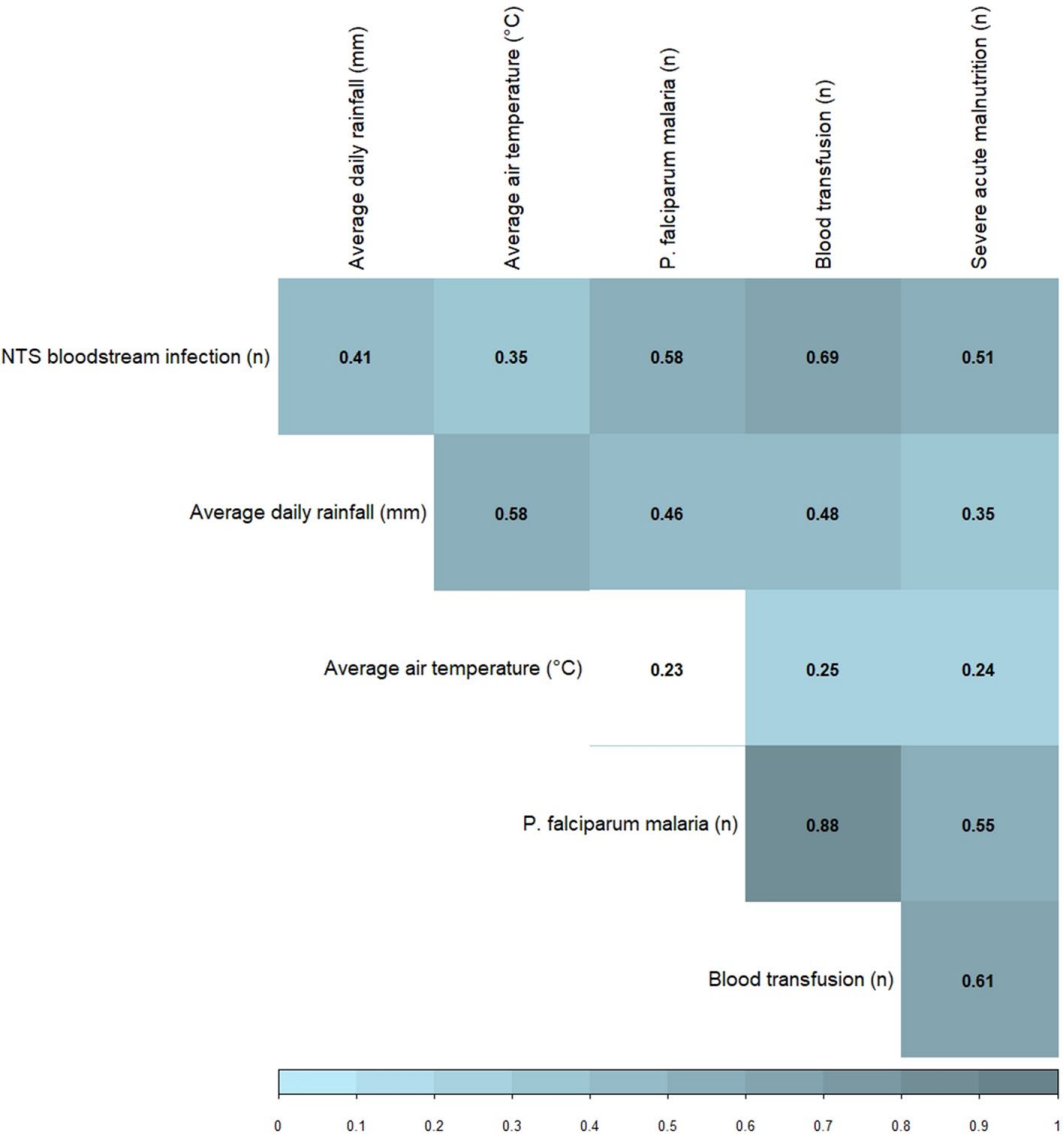
Spearman correlation matrix of association between the monthly number of non-typhoidal *Salmonella* (NTS) bloodstream infections in children under five years old, its environment- factors and host-associated factors (n months = 72, from which two months without data available for severe acute malnutrition). Only the correlation between temperature and *Plasmodium (P.) falciparum* malaria was not significant (p-value > 0.05, non-colored box).

The number of NTS bloodstream infections correlated positively and significantly with both average daily rainfall and air temperature. The positive correlation between NTS and its host-associated factors was stronger than the correlation with its environment-associated factors (Fig. 3). Both rainfall and temperature correlated positively and significantly with all three host-associated factors, demonstrating the importance of disentangling these factors in an explanatory model for NTS dynamics.

### Environment- and host-associated factors were disentangled by principal component analysis

Both environment-associated factors (average daily rainfall and average air temperature per month), all three host-associated factors (*Pf* malaria infections, blood transfusions and children with severe acute malnutrition per month) and both long term trend factors (twelve-month moving average of total blood cultures sampled and total malaria microscopy examinations) were transformed to five relevant and uncorrelated principal components that together explained 98% of the variance of all original variables. The number of *Pf* malaria infections and transfusions loaded mainly on the same component, as did both trend factors. All other components corresponded with a single original variable and explained minimum 76% of the variance of its original variable(s). This facilitated interpretation of the components, which are henceforth named after the original variables they represent. Cross-loading of all host-associated factors accounted for 4.1% and 1.8% of the explained variance of the rainfall and temperature component, respectively (Table 1). A timeseries comparison of the original variables and their respective principal components is shown in Supplementary Fig. S2.

**Table 1:**
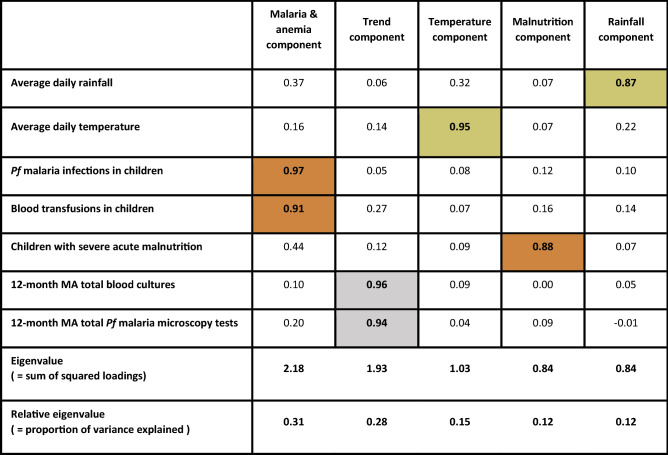
This table summarizes the loading of the environment- (green) and host-associated factors (red) on their respective components after principal component analysis with orthogonal (quartimax) rotation to resolve multicollinearity.

To adjust for the increasing trend in the number of sampled children over the years, 12-month moving averages of total blood cultures and malaria microscopy examinations (light grey) were included in the principal component analysis. Abbreviations: MA, moving average; *Pf*, *Plasmodium falciparum.*

### Multivariable modelling of NTS and its environment- and host-associated factors

**Figure 4:**
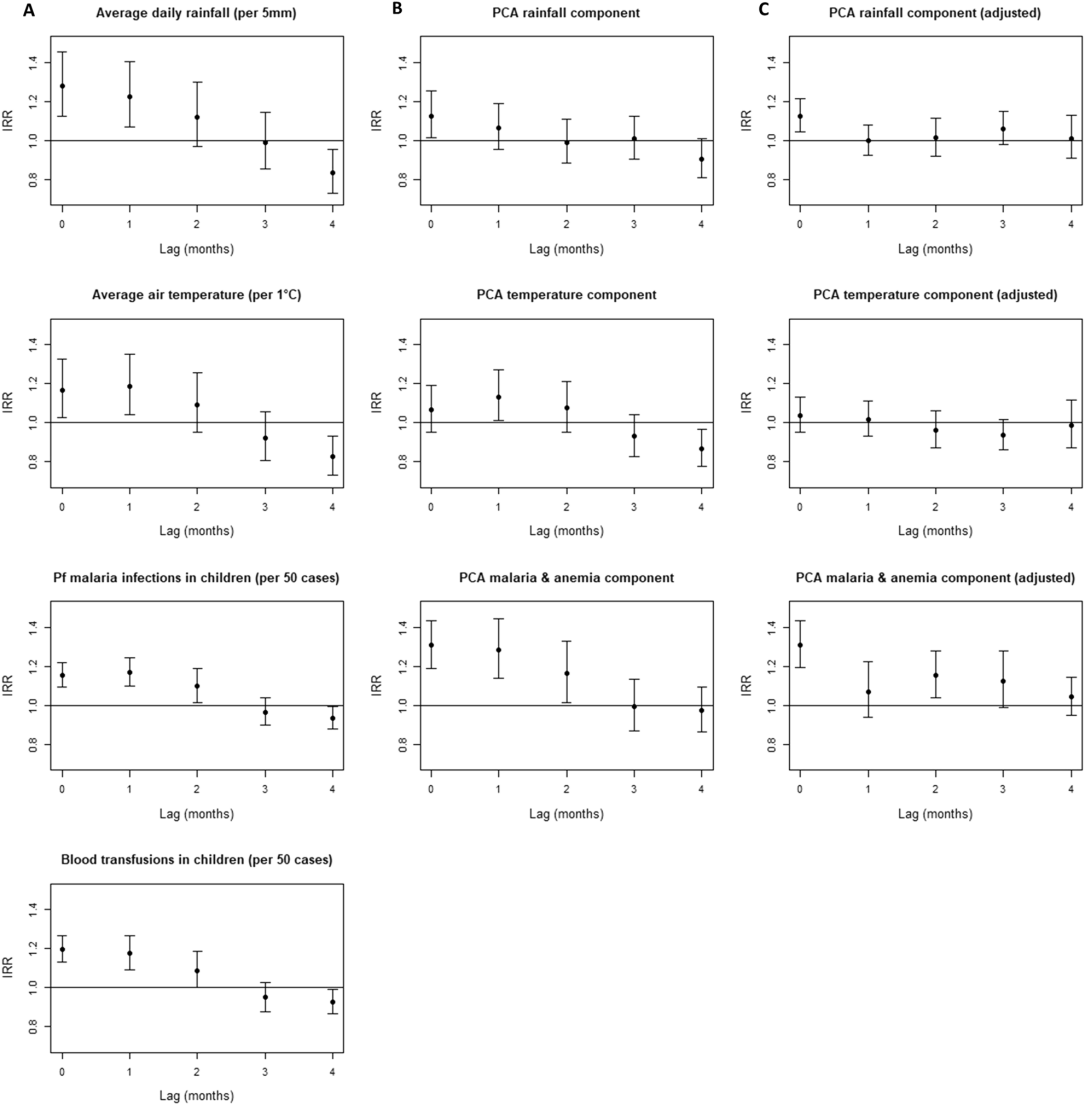
Association of environment- and host-associated factors with non-typhoidal *Salmonella* (NTS) bloodstream infection in young children at different time points (0 up to 4 months lagged). Incidence rate ratios (IRR) were calculated with negative binomial regression. (**A**) Association between original environment- and host-associated factors and NTS bloodstream infections, adjusted for autoregression and for increasing trend in total blood cultures sampled and malaria microscopy examinations. (**B**) Association between principal components representing the environment- and host-associated -factors, adjusted for autoregression and for the principal component representing the increasing trend in total blood cultures sampled and malaria microscopy examinations. (**C**) Association between principal components representing the environment- and host-associated factors, adjusted for the environment- and host-associated components retained in the final model (non-lagged rain, non-lagged malaria & anemia, 2 months lagged malaria & anemia and non–lagged malnutrition component), for autoregression and for the principal component representing the increasing trend in total blood cultures sampled and malaria microscopy examinations. Abbreviations: IRR, Incidence Rate Ratio; *Pf*, *Plasmodium falciparum*.

A positive and significant association between NTS and each non-lagged environment- or host-associated factor was demonstrated after adjustment for autoregression and the increasing trend in blood cultures and malaria examinations (Fig. 4A). Each 5 mm increase in average daily rainfall explained a 1.28 factor increase in number of NTS infections that month and each degree Celsius increase in average air temperature a 1.16 factor increase. Each increase of 50 *P. falciparum* malaria infections or blood transfusions explained a 1.16 or 1.20 factor increase in number of NTS infections that month. Each additional case admitted with severe acute malnutrition explained a 1.02 factor increase in number of NTS infections that month (95% CI 1.01–1.03). For lagged environment- and host-associated factors, a turning point was observed at three months lag with a loss of positive association with NTS (Fig. 4A).

When the same analysis was repeated with principal components instead of the original environment- and host-associated factors and trend variables, the pattern of associations differed. The non-lagged rainfall (IRR = 1.28, 95% CI 1.13–1.45) and malaria & anemia component (IRR = 1.31, 95% CI 1.19–1.43) were still significantly and positively associated with NTS. However, the positive association with temperature (IRR = 1.06, 95% CI 0.95–1.19) and malnutrition component (IRR = 1.08, 95% CI 0.97–1.21) lost statistical significance. For the lagged temperature and malaria & anemia components, the turning point with a loss of positive association was still observed at three months lag, while for the lagged rainfall components, the turning point shifted to 2 months lag (Fig. 4B). All lagged components that were positively associated with NTS, *i.e.* with fewer months lag than the turning point, were included in the full model used for step forward selection of the final model.

Full model:

$$\begin{aligned} \log \left( {\mu_{t} } \right) = & \beta_{0} + \beta_{1} \log \left( { Y_{t - 1} + 1} \right) + \beta_{2} \;trend\;component _{t} + \beta_{3} \;rain\;component_{t} \\ & \quad + \beta_{4} \;rain\;component _{t - 1} + \beta_{5} \;temperature\;component_{t} \\ & \quad + \beta_{6} \;temperature\;component_{t - 1} + \beta_{7} \;temperature\;component_{t - 2} \\ & \quad + \beta_{8} \;malaria\;\& \;anemia\;component_{t} + \beta_{9} \;malaria\;\& \;anemia\;component_{ t - 1} \\ & \quad + \beta_{10} \;malaria\;\& \;anemia\;component_{t - 2} + \beta_{11} \;malnutrition\;component_{t} \\ \end{aligned}$$

The final model identified with multivariable step forward selection included the trend component and autoregression term, the non-lagged rainfall component, the non-lagged and two-months lagged malaria & anemia component and the non-lagged malnutrition component.

Final model:

$$\begin{aligned} \log \left( {\mu_{t} } \right) = & \beta_{0} + \beta_{1} \log \left( { Y_{t - 1} + 1} \right) + \beta_{2} \;trend\;component _{t} + \beta_{3} \;rain\;component_{t} \\ & \quad + \beta_{4} \;malaria\;\& \;anemia\;component _{t} + \beta_{5} \;malaria\;\& \;anemia\;component_{ t - 2} \\ & \quad + \beta_{6} \;malnutrition\;component_{t} \\ \end{aligned}$$

**Figure 5:**
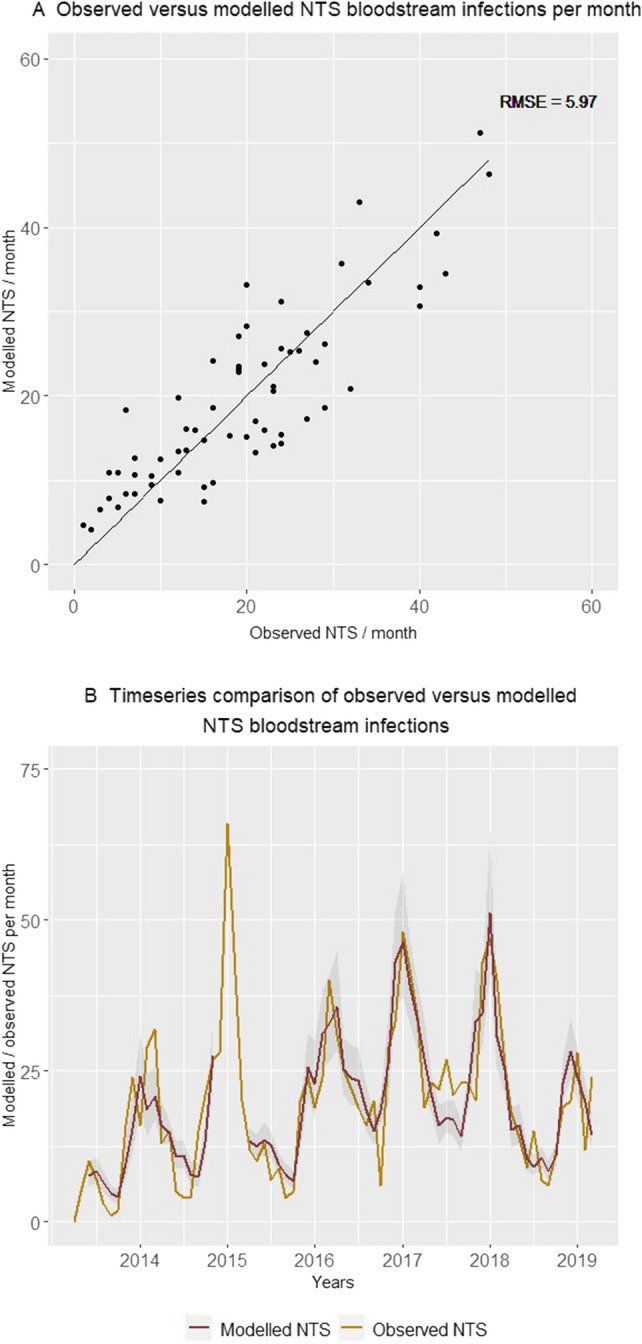
Comparison of the number of non-typhoidal *Salmonella* (NTS) bloodstream infections in children under five years old admitted to Kisantu general referral hospital that were observed and modelled by the final model per month. In panel A, pairs of observed and modelled NTS infections are displayed. In Panel B, the observed (gold) and the modelled (red) number of NTS infections with the 95% confidence interval of modelled NTS infections (grey) are displayed as a timeseries. The gap (December 2014–May 2015) in the modelled number of non-typhoidal *Salmonella* bloodstream infections is caused by missing data for the number of children with severe acute malnutrition admitted to the nutritional unit from December 2014 till February 2015. Abbreviations: NTS, non-typhoidal *Salmonella*; RMSE, Root Mean Square Error.

All were positively associated with NTS and this association was statistically significant for all variables (Table 2). The final model explained 75% of monthly NTS variation. Differential comparison of the generalized R2 of models by sequential addition of components revealed that addition of host-associated components to the basic model explained an additional 17.5% of monthly NTS variation. Further addition of rainfall to the model with host-associated components explained an additional 9% of monthly NTS variation (Table 2). All IRR estimated by the final model corresponded reasonably well with the ones of the full model, which indicated model stability (Supplementary Table S1). In addition to the model parameters in Table 2, the goodness of fit can be visually assessed in Fig. 5, in which the observed and modelled NTS bloodstream infections are compared. Figure 4C confirms that, when controlled for the variables retained in the final model, none of the other non-lagged and lagged environmental and host-associated components were significantly associated with NTS. Multicollinearity (variance inflation factors in Supplementary Table S2) and autocorrelation (partial autocorrelation plot in Supplementary Fig. S3) were adequately resolved in the final model.

**Table 2:** A model of environmental and host-associated components explains the seasonal dynamics of non-typhoidal *Salmonella* (NTS) bloodstream infections in children < 5 years old admitted to Kisantu hospital, DR Congo.

Final model: n months = 66 AIC = 433; Ɵ = 21.6	Incidence rate ratio (95% CI)	*P*-value	Generalized R2		
Rain component	1.13 (1.04–1.21)	0.002	+ 0.09	0.66	0.75
Severe acute malnutrition component	1.09 (1.01–1.17)	0.04	+ 0.01		
*Pf* malaria & anemia component (lag 2 months)	1.16 (1.05–1.29)	0.003	+ 0.08		
*Pf* malaria & anemia component	1.30 (1.20–1.41)	< 0.001	+ 0.09		
Trend component	1.22 (1.09–1.36)	< 0.001	0.48		
Autoregression	1.34 ( 1.12–1.61)	0.001			
Intercept	7.34 (4.34–12.39)	< 0.001			

AIC: Akaike Information Criterium; Generalized R2: coefficient of determination as specified by Zhang D. that indicates the proportion of variation explained^14^, data shown were rounded to facilitate interpretation; 95% CI, 95% Confidence Interval; *Pf, Plasmodium falciparum;* Ɵ, dispersion parameter.

## Discussion

The present study confirmed the increased occurrence of NTS bloodstream infections during the rainy season in hospital admitted children under five years old in a tropical setting endemic for NTS bloodstream infections and *Pf* malaria. Most importantly, this study revealed a direct association between rainfall and NTS seasonal dynamics, independent of seasonal variation in host-susceptibility. While host susceptibility explained 17.5% of NTS seasonal dynamics, rainfall alone explained an additional 9% of monthly NTS variation. Seasonal variation in host susceptibility occurred, as demonstrated by the association between the number of NTS bloodstream infections on the one hand and the numbers of (i) *Pf* malaria infections & blood transfusions and (ii) children with severe acute malnutrition on the other hand. There was no evidence for delayed effects of environment-associated factors, in contrast to the up to two months delayed association between *Pf* malaria & anemia cases and NTS bloodstream infections.

Many studies previously reported the predominance of NTS bloodstream infections during the rainy season and concurrence with peaks in *Pf* malaria and malnutrition, although the exact timing within the rainy season varied^15–25^. Only two other studies assessed climate influence on NTS bloodstream infections in sub-Saharan Africa; both occurred in Blantyre in Malawi and were partially based on the same dataset^9,17^. The first study in Malawi from Feasey et al. demonstrated, based on structural equation modelling, that rainfall was only indirectly related to NTS through its impact on malaria and malnutrition. The different conclusions of the study from Feasey et al. compared to the present one may be explained by the urban setting, higher HIV prevalence and lower malaria prevalence^17^. The second study in Malawi from Thindwa et al. did not take into account host-associated factors and should therefore be interpreted with caution. In the study from Thindwa et al., both immediate and delayed associations between rainfall and NTS were demonstrated^9^. In addition, Thindwa et al. reported delayed positive associations between temperature and NTS at lower temperatures (< 19 °C), but negative associations between temperature and NTS at higher temperatures (> 28 °C). These observations do not match with environmental *Salmonella* proliferation and survival, because *Salmonella* proliferation is ideal at 35–37 °C and growth inhibition occurs at temperatures below 15°C^26^. The absence of a clear association between temperature and NTS in our study may be explained by the limited variability in average air temperature, which may be insufficient to cause biological variation in environmental NTS survival and proliferation. Secondly, in our study, non-linear effects were not modelled. Finally, average satellite-derived air temperature might not adequately reflect the conditions of the environmental niche of NTS, *e.g.* temperature in soil or water reservoirs.

The direct association between rainfall and NTS observed in our study can reflect environmental, behavioral and biological drivers. If rainfall effectively promotes NTS transmission, the absence of delayed association between rainfall and NTS in the present study points to a short-cycle transmission with a short incubation period^9^. Rainfall has indeed the potential to act as an environmental driver and facilitate waterborne NTS transmission. In DR Congo, about half of the households use unimproved sanitation facilities (uncovered latrines or open defecation) and unimproved water sources (surface water or unprotected wells and springs)^27,28^. Flooding and water runoff caused by heavy rains may contaminate these water sources with human or animal feces^8^. Previous studies in the Gambia and Ghana reported that unprotected wells were most fecally contaminated during the rainy season and NTS were isolated sporadically from these wells^29,30^. However, in a study in Burkina Faso, no NTS were cultured from the main water source of the households of children with NTS bloodstream infection^6,31^.

In addition to waterborne environmental transmission, environmental transmission via contaminated crops and soil is possible, *e.g.* via splashes of contaminated water on green leaves^8^. However, the internalization of NTS in crops can take time^8^ and the harvest and consumption of the crops might not be immediate. Therefore, given the absence of a delayed rainfall association, crop-mediated transmission might be less likely^9^. However, farming activities, food availability and consumption vary seasonally, may act as seasonal environmental and behavioral drivers of NTS transmission and confound the association between rainfall and NTS^32^. For example, in the present study area, groundnuts and fruits are harvested and consumed in the early rainy season, while cassava is eaten all year round^32^. If these foods were more frequently contaminated or carried a higher risk of infection, they might have altered NTS seasonal dynamics. In addition, more indoor activities during wet months may increase person-to-person transmission^9^. Finally, if an animal reservoir would be implied, seasonally influenced animal behavior and reproduction might also affect NTS transmission^8,33^.

The present study confirms that seasonal variation in host susceptibility is an important driver of NTS seasonal dynamics. *Pf* malaria infections predispose to NTS bloodstream infection by impairment of the gastro-intestinal mucosal barrier, by macrophage dysfunction and by suppressed antibody responses^34^. In addition, red blood cells are destroyed during *Pf* malaria infection, which causes severe hemolytic anemia. Severe hemolysis further impedes protection to NTS infection and blunts normal neutrophil and macrophage defense mechanisms^34^. Both current and recently cured *Pf* malaria infections increase the risk of NTS bloodstream infection and may contribute to the up to two months delayed association between *Pf* malaria and NTS observed in this study^34^. In addition, repeated (a)symptomatic *Pf* malaria infections in young children cause chronic hemolytic anemia and may explain the latter delayed association^35^. Hemoglobinopathies such as sickle cell disease are another frequent cause of chronic hemolytic anemia in this setting and may aggravate splenic dysfunction and impairment of the gastro-intestinal mucosal barrier^36,37^. Malnutrition is the last well-known and seasonally fluctuating host-associated risk factor for NTS due to the altered gastro-intestinal barrier, impaired serum complement activity, impaired macrophage and neutrophil functions, and reduced B-cell numbers^2,38^. Other seasonally variable biological factors may theoretically alter host susceptibility, but have, to our knowledge, not been described yet^2^.

This retrospective study had some limitations. Firstly, strong multicollinearity in the raw data complicated the analysis. We resolved multicollinearity, before regression analysis, by principal component analysis with orthogonal quartimax rotation. Minimal collinearity between the principal components was confirmed by the high unique component loadings (Table 1) and by the low variance inflation factors of all components retained in the final model (Supplementary Table 2). Missing data occurred, but were limited by the use of systematically reported hospital data for host-associated factors and freely available remote sensing data for environment-associated factors. Nevertheless, even when hospital registers appeared to be complete, random occurrences may have missed. Moreover, the sparsity of ground-based weather observations in sub-Saharan Africa hamper validation and fine tuning of remote sensing data^39,40^. In addition, the present data were spatially aggregated to cover the entire Kisantu district, but did not allow to consider local differences in rainfall and temperature and their impact on NTS seasonal dynamics. Temporal averaging of rainfall and temperature data impeded the study of extreme weather events and we did not take into account non-linear relationships. This study is also limited in that it is a single center hospital-based study. The hospital-based number of NTS bloodstream infections and the hospital-based data used to model host-associated factors probably underestimated the community incidence of NTS, *Pf* malaria, anemia and malnutrition due to limited health care utilization, self-medication, non-referral and low sensitivity of blood cultures. However, due to its flat fee system for referred patients, Kisantu hospital is frequently consulted. Moreover, a blood culture is routinely sampled in each child with severe febrile illness and processed free of charge, which resulted in high surveillance coverage and a large NTS collection representing also the more vulnerable patients of population in a cost-effective way^5^. Finally, bad road conditions due to heavy rainfall may have impacted health care seeking behavior during the rainy season and flattened the relation between rainfall and NTS occurrence^5^.

The current findings should be confirmed in other geographical and epidemiological settings, *e.g.* in regions with seasonal hyperendemic *Pf* malaria or at community level. In addition, we recommend to assess if similar models can be used for NTS prediction and outbreak detection^24,41,42^. The direct contribution of rainfall to environmental NTS transmission should be further confined by case–control and environmental sampling studies. Integration of a spatial variability, *e.g.* in relation to water drainage and proximity to water bodies, in the current model approach may aid in identifying the sites and period of interest for environmental sampling. Last, but not least, the causal contribution of environmental drivers to NTS seasonal dynamics, be it directly or indirectly, should be elucidated to estimate the impact of environmental changes, such as deforestation and urbanization, and climate change. Although effects of climate change will vary regionally in Africa, warming, drought and extreme weather events are expected and may have the potential to increase disease transmission and vulnerability^43,44^.

In conclusion, this study provided evidence for a direct epidemiological association between rainfall and NTS bloodstream infections, independent of seasonal variation in host-susceptibility. This direct association between rainfall and NTS may reflect an environmental cycle in NTS transmission that should be confirmed with environmental studies. In addition, the seasonal increase in host susceptibility for NTS bloodstream infection caused by *Pf* malaria, anemia and malnutrition was confirmed. Local environmental and global climate change have the potential to increase the NTS burden in children, either directly or by altering host susceptibility. This potential impact stresses the importance of NTS prevention by water, sanitation and hygiene (WASH) interventions, malaria control programs and NTS vaccine deployment^2,3^.

## Methods

### Study site and period

In this study, retrospective data from St. Luc Kisantu general referral hospital (Kisantu hospital) from April 2013 to March 2019 were assessed. Kisantu hospital provides health services to Kisantu health district (locally referred to as health zone), which is situated 120 km south of Kinshasa in the province Kongo Central (DR Congo; Fig. 1). Kisantu health district has a surface of approximately 1,400 km^2^ and is subdivided in 5 semi-urban health areas surrounding Kisantu hospital and 12 more remote rural health areas. The population of Kisantu health district increased from approximately 168,000 inhabitants in 2013 to 202,448 inhabitants in 2019. Almost half of them were living in the semi-urban health areas and the latter represented the great majority of patients admitted to Kisantu hospital. Kisantu hospital has about 340 beds and has a flat-fee system for patients referred by health centers that are located in the health areas, which results in high bed occupancy rates, *e.g.* of 144% in the pediatric ward in 2017^12^.

In Kisantu health district, the burden of NTS bloodstream infections is high, particularly in young children^5,42,45,46^. In addition, *Pf* malaria is holoendemic and 24% of children (6–59 months) in Kongo Central had a positive blood microscopy test during a national health survey in 2013–2014^27^. Moreover, 69% of children (6–59 months) in Kongo Central were anemic and 11% of children under five years old were suffering from acute malnutrition^27^. HIV-prevalence in Kongo Central is relatively low with 0.2% of the adult population infected^27^.

Kongo Central province is situated in the humid tropics. The local climate regime is characterized by one distinct dry season from June to September and a rainy season from October to May, with an intermittent decrease in precipitation in January and February (Fig. 2B)^47^. Kisantu district is characterized by a hilly topography, with altitudes ranging between 400 and 700 m above sea level^48^. The average annual rainfall is 1000–2000 mm and the annual average temperature varies around 25 °C with markable lower temperatures during the dry season (Fig. 2B, C)^47^.

### Data collection

#### Blood culture surveillance

Upon admission to Kisantu hospital, one blood culture (1–4 ml blood in BacT/ALERT bottles (bioMérieux, Marcy-L’Etoile, France) was routinely sampled from each child suspected of having a bloodstream infection, as previously described^5,45,46,49^. Free-of-charge blood culture sampling and work-up started in 2007 at Kisantu hospital, as part of the blood culture surveillance network coordinated by the Institut National de Recherche Biomédicale (Kinshasa, DR Congo) and the Institute of Tropical Medicine (Antwerp, Belgium)^5,45,46,49^, and was fully integrated in routine clinical care by 2013. Indications for blood culture sampling and blood culture sampling work-up remained consistent over the years. Only blood cultures sampled from children older than 28 days and younger than 5 years were taken into account for analysis. The number of total blood cultures sampled per month was defined as the number of blood cultures sampled from this age group. The number of NTS bloodstream infections per month was defined as the number of blood cultures from which NTS was isolated and identified based on biochemical tests and serotyping^5,45,46,49^.

#### Hospital statistics of host-associated factors

Retrospective data from hospital registers were used as proxy variables for the monthly occurrence of clinical conditions associated with an increased risk of NTS bloodstream infection in young children. The number of *Pf* malaria infections per month in hospital admitted children under five years old was modelled as the number of positive malaria microscopy examinations per month in children < 14 years old. Likewise, the total number of malaria microscopy examinations per month was counted as the monthly number of positive and negative microscopy examinations in children < 14 years. Diagnosis of severe acute malnutrition and severe anemia requiring blood transfusion were made according to the WHO criteria^50^. The monthly number of transfused blood bags was used as a proxy for the monthly number of admitted children under five years old with severe anemia requiring blood transfusion. The number of children admitted with severe acute malnutrition to the nutritional unit in the pediatric ward of Kisantu hospital was used as proxy for children under five years old admitted with severe acute malnutrition. HIV was not modelled, because HIV is a less important risk factor for NTS bloodstream infections in children under five years old in DR Congo^5,27^ and does not vary seasonally.

#### Remote sensing of environment-associated factors

In the absence of an on-site weather station, rainfall and temperature were estimated by satellite-based remote sensing techniques^51^. Remote sensing products were chosen based on their temporal and spatial resolution; data availability covering the full study period and free availability on Google Earth Engine^12^. For rainfall, the product “Climate Hazards Group InfraRed Precipitation with Station Data” (CHIRPS) daily version 2.0 was chosen^52^, because it combines infrared satellite imagery at 0.05° resolution with in-situ station data. For temperature, the parameter “2 m air temperature” was extracted from the ERA5 DAILY dataset, a standard remote sensing temperature dataset which represents the daily average air temperature at 2 m above the surface with a spatial resolution of 0.25°^53^. The extracted daily rainfall and temperature data were spatially averaged over the health district and aggregated per month to obtain the average daily rainfall per month, minimum and maximum daily rainfall per month and the average air temperature per month.

### Statistical analysis

#### Descriptive statistics

Data analysis was done in R version 3.6.1. The dataset and R-script are available at 10.6084/m9.figshare.14783850 and 10.6084/m9.figshare.14784318, respectively. Monthly data were plotted over time and per month to visualize seasonal dynamics. Spearman’s rank correlation coefficients were calculated between the monthly number of NTS bloodstream infections, host-associated and environmental factors.

#### Timeseries regression model development

To resolve multicollinearity between environment- and host-associated factors and facilitate interpretation of each component, principal component analysis with quartimax rotation was performed using the R-package “psych”^54^. The sixth component contributed < 3% of remaining variance and did not represent an associated factor, and was therefore discarded in further analysis.

To explain the short-term seasonal variation in the monthly number of NTS bloodstream infections, a time series regression was performed^55,56^. Multivariable timeseries regression models were built according to the following formula:

1 $$Y_{t} \sim {\text{NegBin }}\left( {\mu_{t} , \psi } \right)$$

2 $$\log \left( {\mu_{t} } \right) = \beta_{0} + \beta_{1} \log \left( { Y_{t - 1} + 1} \right) + \beta_{2} x_{t} + {\Sigma }\beta_{k} z_{p,tlag 0 - 4} + \varepsilon_{t}$$

Negative binomial regression with mean ${\mu }_{t}$ and dispersion parameter $\psi$ Eq. (1) was chosen to model the overdispersed count outcome data (R-package “MASS”; overdispersion tested with Pearson Chi2 dispersion statistic, *P* < 0.05)^55–57^. Potential long term trends that stem from an increasing number of hospital admissions in children–rather than environment- or hospital-associated factors—were accounted for by the introduction of the moving average of total blood cultures sampled and total malaria microscopy examinations or their corresponding principal component of the previous 12 months (x_t_). The infectious nature of NTS bloodstream infections implies the presence of temporal autocorrelation. This was accounted for by the autoregressive term log (Y_t-1_ + 1), because the logarithmic scale is a more likely reflection of NTS transmission^56^. To model the relation between the number of NTS per month and the monthly environment- and host-associated factors, the following factors or their respective principal components were modelled (z_p,t_): average daily rainfall per month, average air temperature per month, number of positive malaria microscopy tests per month, number of blood transfusions per month and number of children admitted with severe acute malnutrition to the nutritional unit. Except for malnutrition, we modelled delayed effects up to a four month lag of environment- and host-associated factors (z_p,t lag 0–4_), because these are biologically plausible due to long term survival of NTS in the environment^58,59^ and the long term impaired immunity linked to (chronic) malaria and anemia^34,35,60^. The model was summarized in Eq. 2 with *β*_0_ representing the intercept, *β*_1−*k*_ representing all regression coefficients and *ε*_*t*_ representing the residuals. Incidence rate ratios (IRR) were calculated by the R-package “Epi”. To define the variation explained by the final model, a generalized coefficient of determination, *i.e.* generalized R^2^, was calculated based on mean and variance functions according to the method of Zhang et al. This method was chosen to comply with the negative binomial distribution of the present model and was calculated in the R-package “rsq”^14^.

#### Timeseries regression model selection and analysis

Each non-lagged and lagged environment- and host-associated factor was first regressed individually as its original variable while accounting for autoregression and long term trend (Fig. 4A). Next, the non-lagged and lagged environment- and host-associated components were regressed individually, still accounting for autoregression and long term trend (Fig. 4B). In both regressions, lagged terms were regressed in a simple unconstrained lag model^55^. Based on the simple unconstrained lag model of principal components, the non-lagged and lagged environment- and host-associated components that were positively associated with NTS seasonal dynamics were selected to build the full model together with the autoregressive term and long term trend component (Supplementary table S1). Negative associations, albeit significant, were not selected, because they are biologically implausible and were considered as confounded by other (non-) lagged components or the fact that they reflect preceding dry months. The full model and a basic model, *i.e.* NTS bloodstream infections regressed against autoregressive term and long term trend component only, were used to program step forward selection of a final model based on the lowest Akaike Information Criterium (AIC). Finally, each non-lagged and lagged environment- and host-associated component was separately combined with all terms retained in the final model and regressed against NTS to verify their assumed lack of positive contribution to NTS (Fig. 4C).

### Ethical approval

The study complies with the Declaration of Helsinki, the World Health Organization Council for International Organizations of Medical Sciences and international European Centre for Disease Prevention and Control and Clinical and Laboratory Standards Institute guidelines on microbiological surveillance for which no recommendation for an informed consent has been issued. Ethical approval for the microbiological surveillance study was granted and informed consent was waived by the Institutional Review Board of ITM (ref. 613/08), the Ethics Committee of Antwerp University (ref. 8/20/96), and the Ethics Committee of the School of Public Health (Ecole de Santé Publique) of Kinshasa in DRC (ref. 074/2017).

## Supplementary Information

Supplementary Information.

## Data Availability

Dataset:10.6084/m9.figshare.14783850. R-script of the analysis: 10.6084/m9.figshare.14784318. Google Earth Engine Code: 10.6084/m9.figshare.16644661.
