# Supplementary material for: Direct association between rainfall and non-typhoidal Salmonella bloodstream infections in hospital-admitted children in the Democratic Republic of Congo
Source: Sci Rep. 2021 Nov 3;11:21617. doi: 10.1038/s41598-021-01030-x (PMC8566593; doi:10.1038/s41598-021-01030-x)

Direct association between rainfall and non-typhoidal *Salmonella* bloodstream infections in hospital-admitted children in the Democratic Republic of Congo

Bieke Tack[1,2], Daniel Vita[3], Marie-France Phoba[4,5], Lisette Mbuyi-Kalonji[4,5], Liselotte Hardy[1], Barbara Barbé[1], Jan Jacobs[1,2], Octavie Lunguya[4,5], Liesbet Jacobs[6,7]

Affiliations
1: Department of Clinical Sciences, Institute of Tropical Medicine, Antwerp, Belgium
2: Department of Microbiology, Immunology and Transplantation, KU Leuven, Belgium
3: Saint Luc Hôpital Général de Référence Kisantu, Democratic Republic of Congo
4: Department of Microbiology, Institut National de Recherche Biomédicale, Kinshasa, Democratic Republic of Congo
5: Department of Medical Biology, University Teaching Hospital of Kinshasa, Democratic Republic of Congo
6: Department of Earth and Environmental Sciences, KU Leuven, Heverlee, Belgium
7: Ecosystem & Landscape Dynamics, Institute for Biodiversity and Ecosystem Dynamics, University of Amsterdam, Amsterdam, Netherlands

Corresponding author
Bieke Tack

Supplementary information

Figure S1. Timeseries of the yearly moving average of non-typhoidal *Salmonella* (NTS) bloodstream infection in children under five years old and of *Plasmodium falciparum* (*Pf*) malaria infections in children. The yearly moving averages were calculated based on the average number of infections in the 12 previous months.

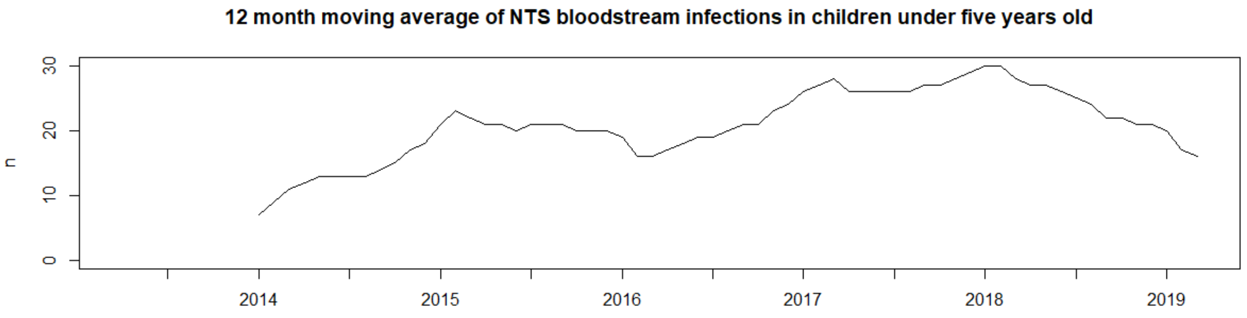

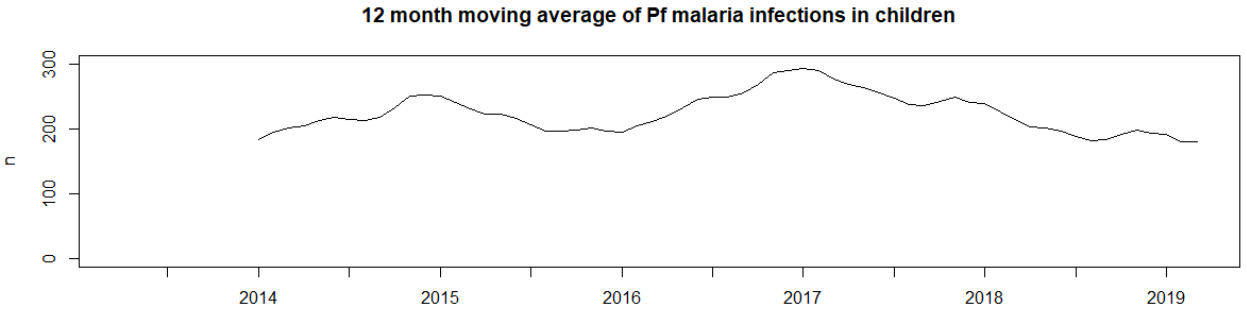

Figure S2. Timeseries comparison of the original independent variables with their respective principal components. Abbreviations: IRR, Incidence Rate Ratio; PCA, Principal Component Analysis; *Pf*, *Plasmodium falciparum*

A. <u>Rainfall</u>

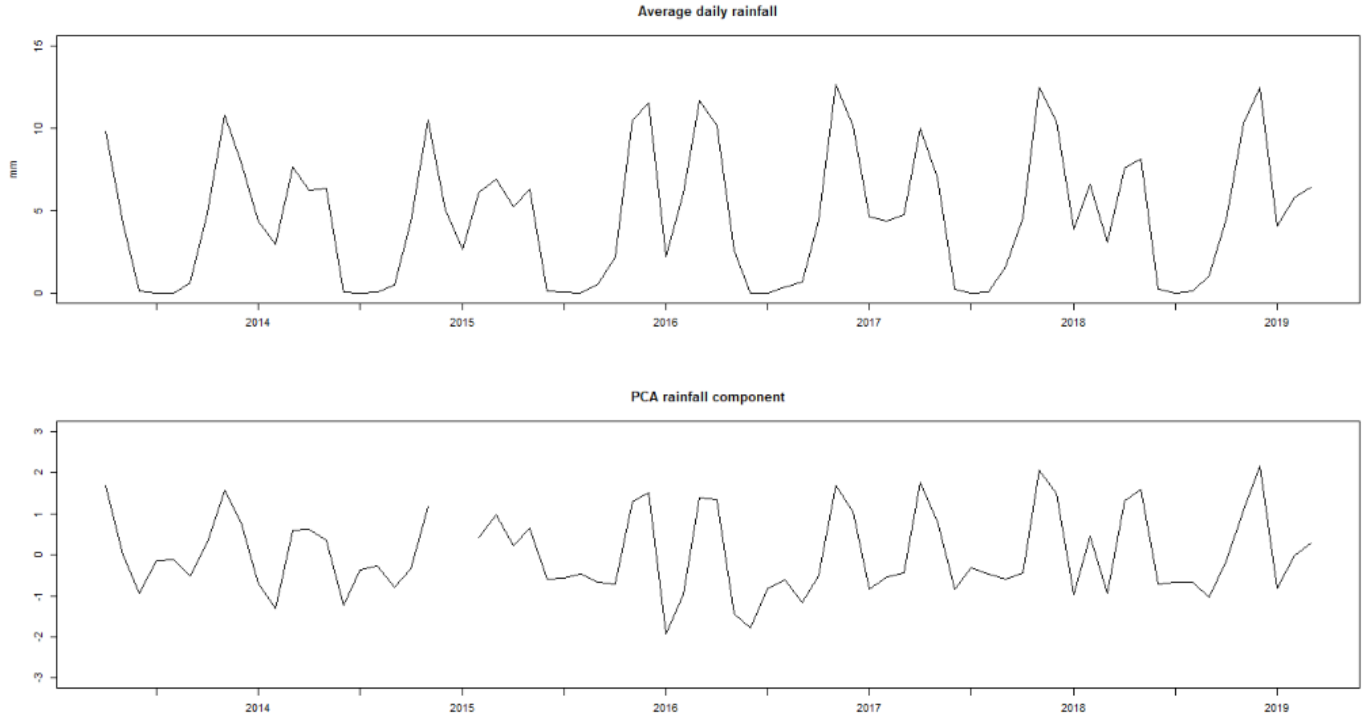

B. <u>Temperature:</u>

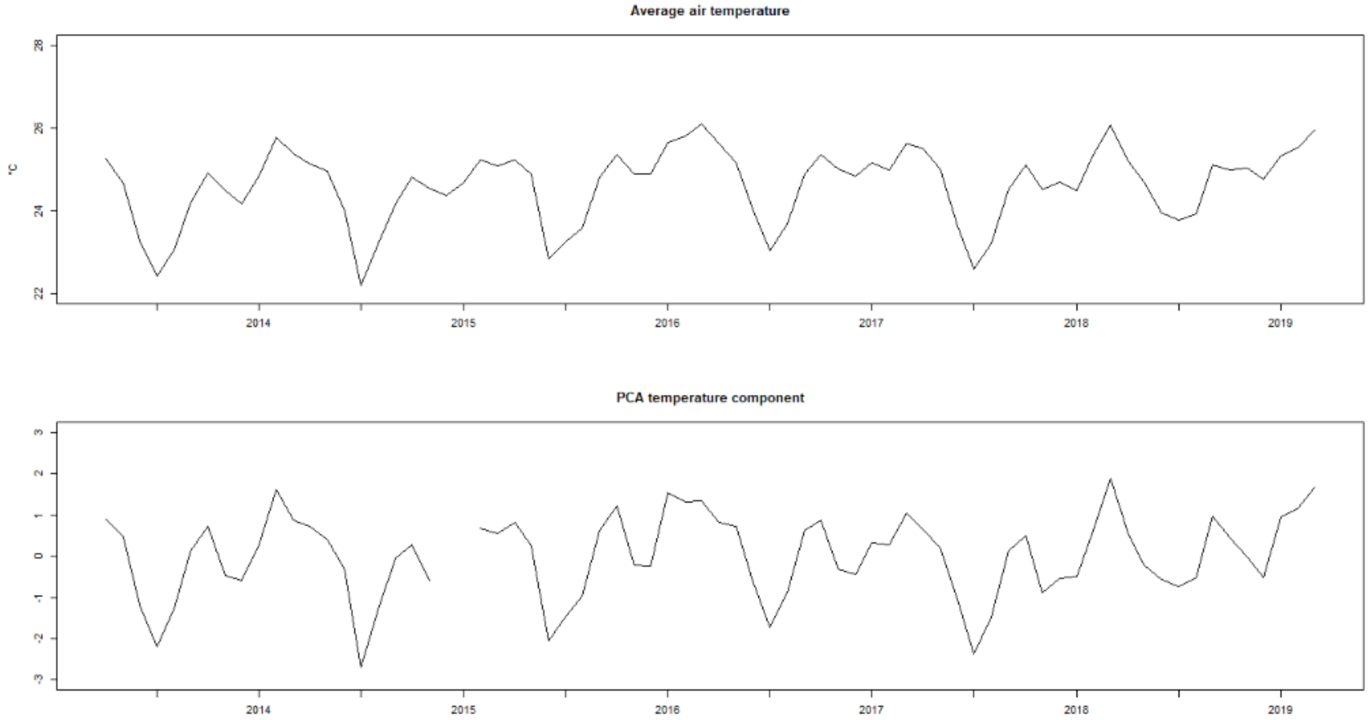

C. Malnutrition:

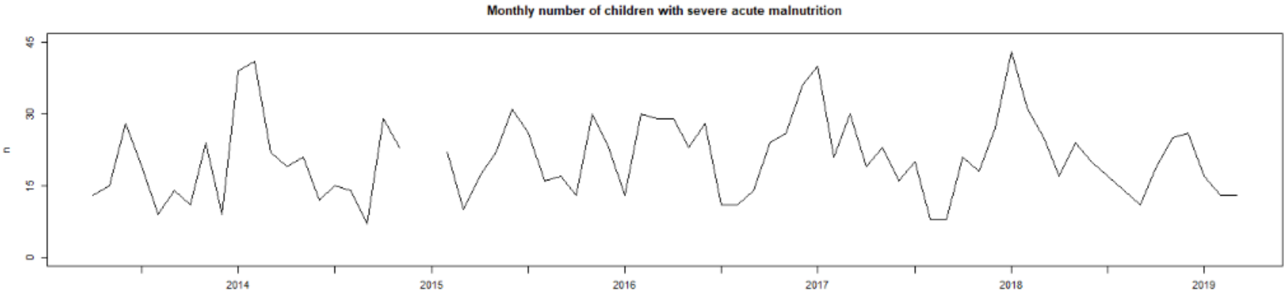

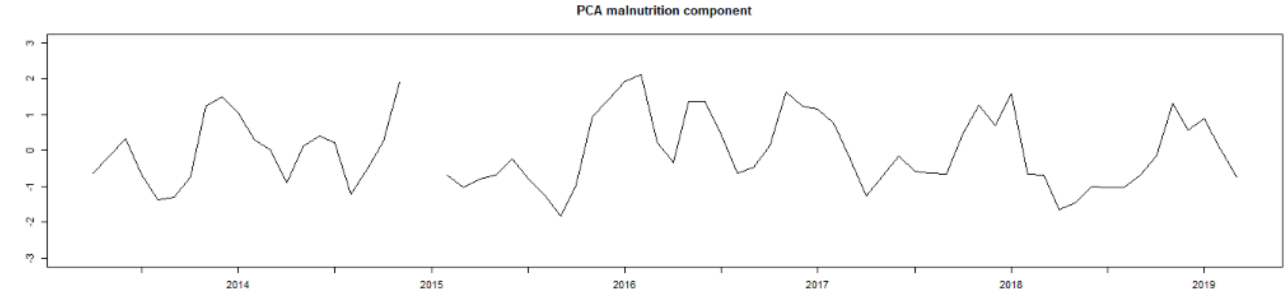

D. *Plasmodium falciparum* malaria and blood transfusions:

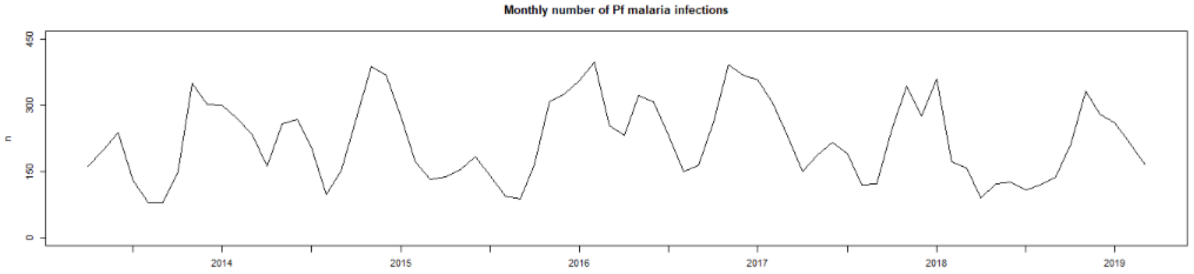

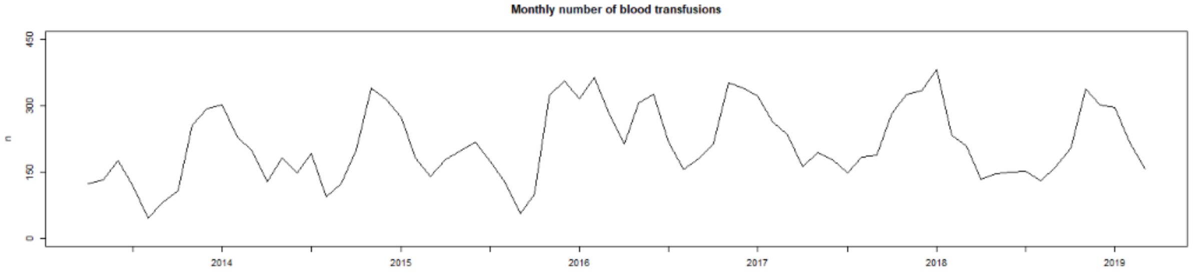

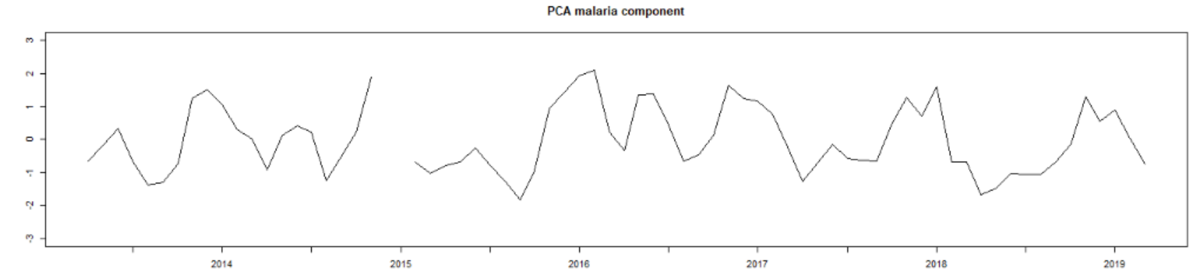

E. <u>Yearly trend in blood cultures and total malaria microscopy examinations:</u>

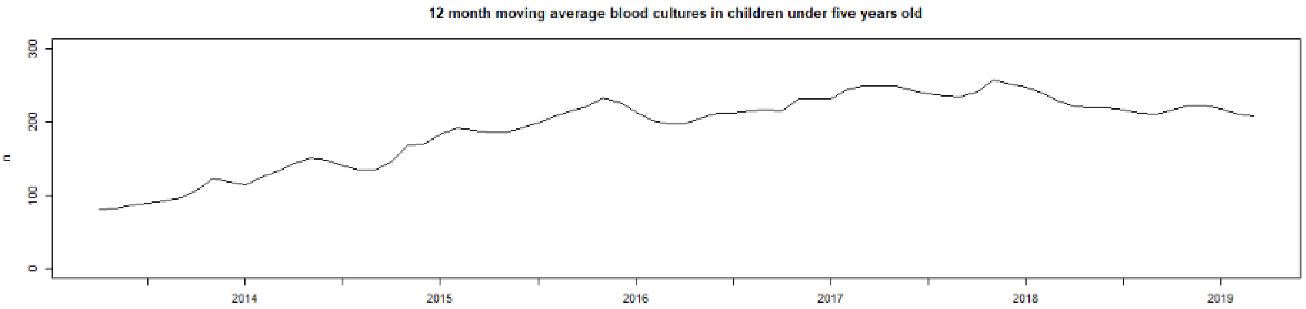

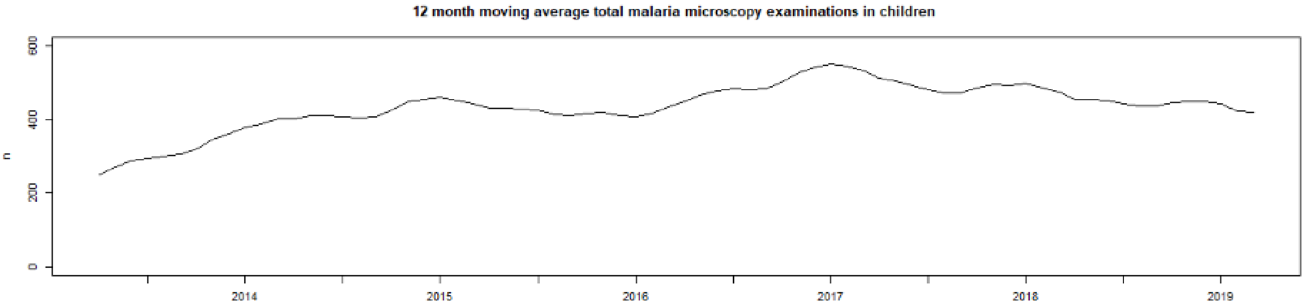

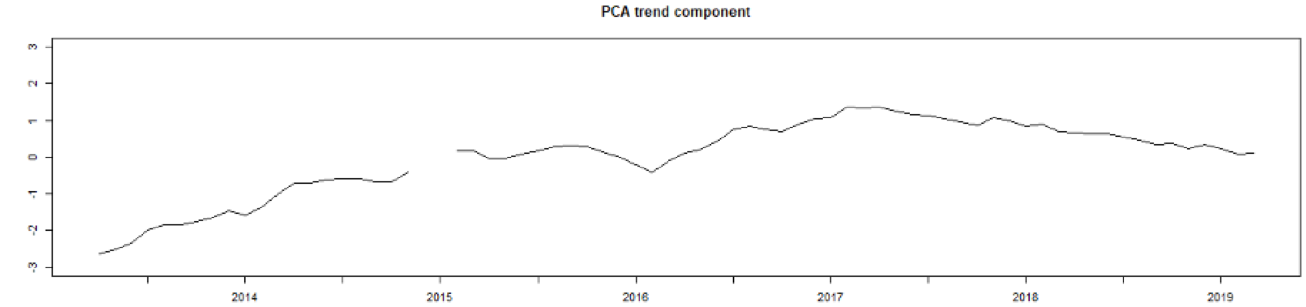

Table S1. Full model of non-lagged and lagged environmental and host-associated components to explain the seasonal dynamics of non-typhoidal *Salmonella* bloodstream infections in children under five years old admitted to Kisantu hospital, DR Congo. Abbreviations: AIC, Akaike Information Criterium; 95% CI , 95% Confidence Interval ; *Pf, Plasmodium falciparum;* Ө, dispersion parameter

Full model: n months = 62<br>*AIC = 442 ; Ө = 21.8*	Incidence rate ratio (95% CI)	p-value
**Rain component**	1.13 (1.05 – 1.22)	0.002
**Rain component (lag 1 month)**	1.00 (0.90 – 1.12)	0.99
**Temperature component**	1.03 (0.90 – 1.18)	0.69
**Temperature component (lag 1 month)**	1.00 (0.83 – 1.21)	0.97
**Temperature component (lag 2 months)**	0.96 (0.83 – 1.12)	0.64
***Pf* malaria & anemia component**	1.33 (1.16 – 1.52)	<0.001
***Pf* malaria & anemia component (lag 1 month)**	0.97 (0.81 – 1.15)	0.69
***Pf* malaria & anemia component (lag 2 months)**	1.17 (1.02 – 1.33)	0.02
**Severe acute malnutrition component**	1.09 (1.01 – 1.18)	0.036
**Trend component**	1.21 (1.07 – 1.35)	0.001
**Autoregression**	1.37 (1.12 – 1.68)	0.002
**Intercept**	6.87 (3.86 – 12.22)	<0.001

Table S2. Variance inflation factors of all variables retained in the final model

Final model	**Variance Inflation Factors**
**Rain component**	1.04
***Pf* malaria & anemia component**	1.11
***Pf* malaria & anemia component (lag 2 months)**	1.59
**Severe acute malnutrition component**	1.09
**Trend component**	1.63
**Autoregression**	2.34

Figure S3. Partial autocorrelation plot of the deviance residuals of the final model

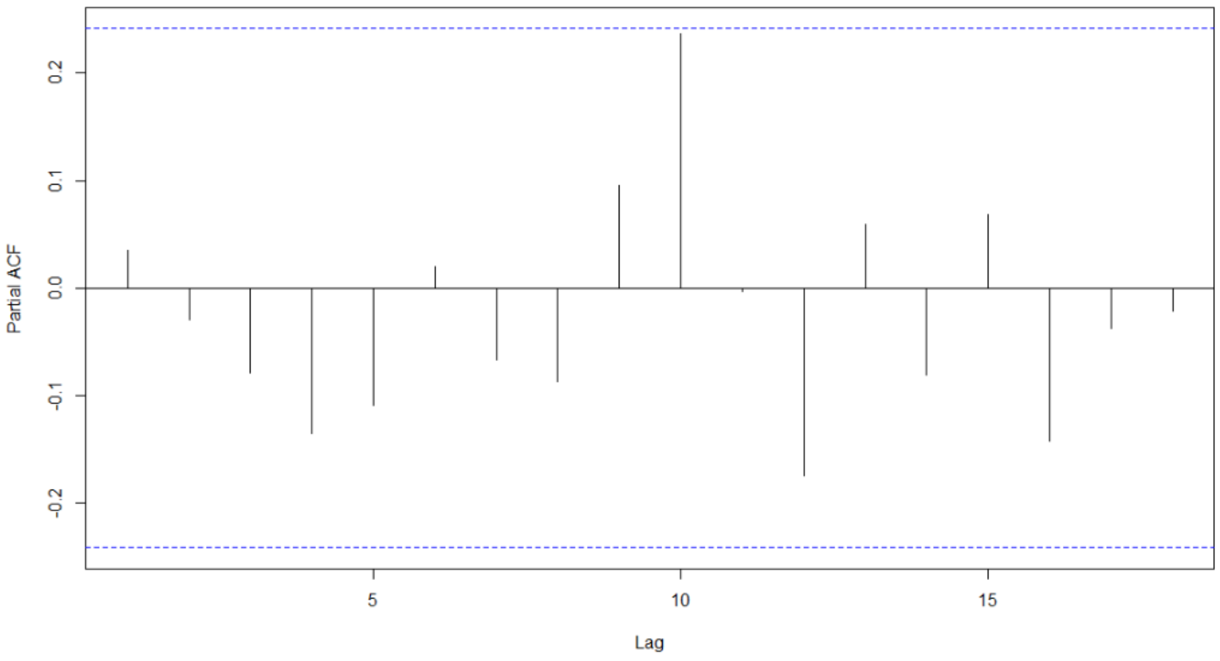

Supplement: Supplementary file 1 — Supplementary Information. [file 41598_2021_1030_MOESM1_ESM.pdf]
